# Molecular adaptation in flowering and symbiotic recognition pathways: insights from patterns of polymorphism in the legume *Medicago truncatula*

**DOI:** 10.1186/1471-2148-11-229

**Published:** 2011-08-01

**Authors:** Stéphane De Mita, Nathalie Chantret, Karine Loridon, Joëlle Ronfort, Thomas Bataillon

**Affiliations:** 1 UMR Diversité, Adaptation et Développement des Plantes Cultivées (DIADE), Institut de Recherche pour le Développement (IRD), Montpellier, France; 2 UMR Amélioration Génétique et Adaptation des Plantes Méditerranéennes et Tropicales (AGAP), Institut National de la Recherche Agronomique (INRA), Montpellier, France; 3 Bioinformatics Research Center, Aarhus University, Denmark; 4 Institute of Biology, Aarhus University, Denmark

## Abstract

**Background:**

We studied patterns of molecular adaptation in the wild Mediterranean legume *Medicago truncatula*. We focused on two phenotypic traits that are not functionally linked: flowering time and perception of symbiotic microbes. Phenology is an important fitness component, especially for annual plants, and many instances of molecular adaptation have been reported for genes involved in flowering pathways. While perception of symbiotic microbes is also integral to adaptation in many plant species, very few reports of molecular adaptation exist for symbiotic genes. Here we used data from 57 individuals and 53 gene fragments to quantify the overall strength of both positive and purifying selection in *M. truncatula *and asked if footprints of positive selection can be detected at key genes of rhizobia recognition pathways.

**Results:**

We examined nucleotide variation among 57 accessions from natural populations in 53 gene fragments: 5 genes involved in nitrogen-fixing bacteria recognition, 11 genes involved in flowering, and 37 genes used as control loci. We detected 1757 polymorphic sites yielding an average nucleotide diversity (pi) of 0.003 per site. Non-synonymous variation is under sizable purifying selection with 90% of amino-acid changing mutations being strongly selected against. Accessions were structured in two groups consistent with geographical origins. Each of these two groups harboured an excess of rare alleles, relative to expectations of a constant-sized population, suggesting recent population expansion. Using coalescent simulations and an approximate Bayesian computation framework we detected several instances of genes departing from selective neutrality within each group and showed that the polymorphism of two nodulation and four flowering genes has probably been shaped by recent positive selection.

**Conclusion:**

We quantify the intensity of purifying selection in the *M. truncatula *genome and show that putative footprints of natural selection can be detected at different time scales in both flowering and symbiotic pathways.

## Background

Understanding the mechanisms underlying adaptation of species to their environment is central to population and evolutionary biology. Recent advances in large-scale sequencing and genotyping now allow rapid characterization of molecular diversity in a set of local populations or across an entire species range but reliably identifying the molecular footprints of adaptation remains challenging [1,2]. Two analytical approaches can be used to identify sequences genes whose evolution has been driven by environment-specific selection. One avenue is to search for genomic regions exhibiting an excess of differentiation between populations, typically using *F*_ST _as a test statistic [3,4]. This approach is most likely to be successful when used in conjunction with a set of populations spanning a clear environmental gradient [5-7] Barring a clear environmental gradient, one can still use allele(s) frequency information, the so-called site frequency spectrum (SFS), often summarized by a unique statistic such as Tajima's *D *to detect departure from selective neutrality [8]. These two methods are based on different aspects of polymorphism data, and thus are expected to be complementary to one another. Moreover, combining the methods may allow for the detection of different forms of selection (i.e. local adaptation and uniform directional selection through a species range) or increasing power to detect more complex selection histories, for example a selective sweep within a single population that can drive an excess of differentiation merely by reducing the level of intra-population diversity. Although genome-wide study of molecular variation and scan for footprints of selection are now routinely reported in major genetical models, candidate gene-centred studies allow for testing of specific hypothesis regarding the evolutionary history of specific functional categories of genes [9].

For plants, timing of flowering is an essential component of fitness. Flowering can be influenced by a variety of climatic factors such as day length, average temperature and drought stress. Accordingly numerous instances of molecular adaptation have been reported for flowering genes, particularly in the annual model plant *Arabidopsis thaliana *[10-13]. The ability of a plant to engage in successful biotic interactions is also integral to fitness. Although numerous work documents the strong imprint of natural selection on plant genes encoding perception of pathogens (so called R genes), both in *A thaliana *but also a suite of other plant species [14-16], much less is known on genes encoding the perception of beneficial (symbiotic) partners. The relative paucity of studies might be explained by the fact that the plant model *A. thaliana *does not engage in symbiosis with any of the two major plant-microorganism symbioses: the ancient and widespread association with mycorrhizae and the more specialized symbiosis with rhizobia.

The barrel medic *Medicago truncatula *is a model legume species used for dissecting the genetics of legume-rhizobia and plant-mycorhizae interactions. Its natural habitat spans the Mediterranean basin. It is an annual selfer found in ecologically unstable populations, resulting in large rates of genetic drift within and among populations [17,18]. For these reasons the efficacy of natural selection to drive adaptive changes is likely to be challenged by genetic drift and population structure.

In this study we examined signatures of natural selection in several genes of two functionally important and largely independent signalling pathways: the flowering and symbiotic pathways in *M. truncatula*. To do this, we surveyed patterns of naturally occurring diversity at the species level for a set of genes in each pathway and a set of control genes. We first quantified the strength of purifying selection in *M. truncatula *and the relative importance of positive selection in genes from both pathways and a set of independent control genes. We then examined broad patterns of genetic subdivision in our sample and split our sample in two ancestral groups. Accordingly, we model demographic factors that influence coalescent history and contemporary levels and patterns of diversity within each group [2]. The rationale for doing so is that strong substructure can blur inference of non-neutral evolution [19]. Finally we searched for signatures of natural selection based on the estimated large-scale population structure, within both ancestral groups with the objective of tracking signatures of selective sweeps in candidate genes.

## Methods

### Plant material

**Figure 1:**
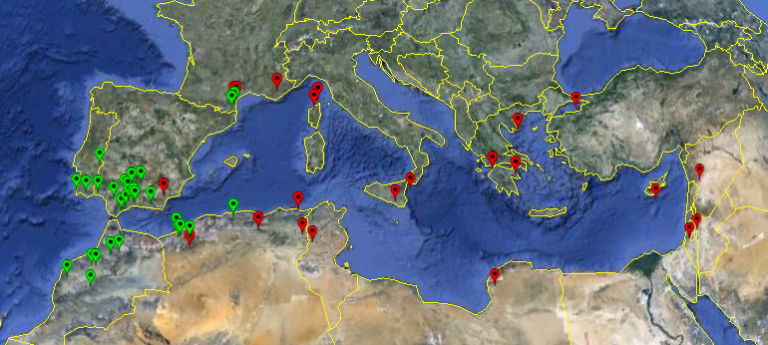
**Geographic origin of the accessions**. Colour-coding refers to the assignation in two groups as inferred using Instruct for *K *= 2. Group 1 (red) comprises 25 accessions and is broadly distributed geographically. Group 2 (blue) consists of *n *= 32 accessions.

A panel of 57 *Medicago truncatula *genotypes (hereafter accessions) consisting of naturally occurring inbred lines sampled from throughout the species range was chosen to represent the species level and a regional level. At the species level, we used a core collection containing 32 accessions, that was previously defined to represent the geographic distribution of the species and to maximize allelic variation at 13 SSR loci initially investigated on a sample of 347 accessions [20]. To investigate genetic diversity at a smaller spatial scale, 25 accessions belonging to a more homogeneous genetic group (as inferred through microsatellite genotyping) were included in the panel. Most of these accessions were initially collected in the Iberian Peninsula and Morocco. The list of accessions used is listed in Additional file 1, Table S1 and their geographic origin depicted in Figure 1. Five other *Medicago *species were included as potential outgroups (one accession per species): *M. tornata*, *M. rigidula*, *M. rigiduloides*, *M. arabica*, and *M. marina*. Unless noted otherwise, all accessions were collected in natural populations and self-fertilized two generations before being grown for DNA extraction.

### Amplification and sequencing

We used data from 37 genic fragments that we PCR amplified and sequenced to model the demographic history of our sample (see below). Primers to amplify these fragments were chosen from two sources [21,22] and within the set of primers available, we selected primers that amplify relatively long fragments (Additional file 2, Table S2). Fragments typically consisted of exon-bound primers spanning one or more introns. The fragment length ranged from 287 to 8897 with a mean of 1644 (Additional file 3, Table S3) and all PCR products were sequenced directly. Protocols for DNA extraction, PCR amplification, fragments purification and Sanger sequencing were identical to those described in De Mita *et al*. [23].

Nodulation gene fragments are located in 5 genes: *DMI1*, *DMI3*, *NIN*, *NFP, PG3*, involved in the Nod factor perception pathway [24]. Primers used for PCR amplification and subsequent sequencing were identical to the ones described in De Mita *et al*. [23]. Flowering gene fragments are located in the following genes: *COL (Constans-Like)*, *FCA2*, *FRLa*, *FTLa*, *FTLb*, *FTLc*, *Soc1b*, *VRN*, *PHYa*, *PHYb*, *PHYe*, which are involved in the signalling pathway controlling day length perception, circadian clock and flowering inception [25]. Two gene fragments, *FCA2 *and *Soc1b*, were amplified from cDNA. For these, total RNA was extracted from fresh leaves using TRI REAGENT™ (T9424, Sigma^®^) buffer and the Reverse Transcription System kit from Promega^®^. Sequences of primers used for PCR amplification and sequencing are available available in Additional file 2, Table S2.

For all gene fragments, sequence reads base calling and annotation were performed manually using Pregap4 and Gap4 programs of the Staden software version 1.6 [26]. Annotation was retrieved either from reference gene sequences when available or from Gene Index Tentative Contigs [27] exhibiting an exact match. All sequences are deposited in Genbank (HQ735420 - HQ738281) and alignments are available from the authors in the Dryad archive (see Availability of supporting data).

### Analysis of patterns of polymorphism and divergence

Analyses of polymorphism were performed using customized scripts in Python and the SeqLib library version 1.6 (S. De Mita and M. Siol, unpublished) incorporating Bio++ version 1.9 [28]. Sequences spanning less than 25% of the length of the longest sequence of the alignment (ignoring all undefined or missing data) were removed. This threshold was raised to 60% for the analysis of synonymous/non-synonymous variation. The *M. tornata *sequence was used as outgroup unless the *M. tornata *sequence was less than 75% of the length of the longest sequence. In these cases, the longest sequence from the other outgroup species was used (see Additional file 3, Table S3). For polymorphism analysis concerning synonymous/non-synonymous variation, estimation of the recombination rate and haplotype analysis, all sites with at least one undefined or missing nucleotide position were excluded. For all other statistics, sites with at least 70% defined data were used.

Counts of the number of sites exhibiting polymorphism and divergence within each gene fragment were used to study the relative roles of positive and purifying selection in shaping polymorphism and divergence in the coding regions. We followed the approach implemented by Welch [29] and recently extended by Obbard *et al*. [9] to infer the fraction 1-*f *of mutation under strong purifying selection and the proportion α of non-synonymous nucleotide divergence driven by positive selection. We used a series of nested models of increasing complexity ranging from the simplistic pure neutrality (*f *= 1, α = 0) to models incorporating a variable intensity of positive or purifying selection (*f *and α are allowed to vary according to each gene category). Estimation of parameters for each gene or category of gene was made using the software MK_test [29]. We used Akaike's Information Criterion (hereafter AIC) to compare models, as suggested by Welch [29]. When models differed by less than 5 AIC units, we used an averaging procedure to obtain robust estimates of α's and f's parameters. Briefly, we computed a model-averaged estimate of α's (and *f*'s) as the weighted average of the individual estimates obtained under each model and used the differences in AIC of each competing model for weighting [30].

### Population structure analysis

We used the Bayesian clustering algorithm implemented in InStruct [31] to analyse population structure. InStruct uses multilocus genotype to assign portions of individual genomes to a predetermined number (K) of clusters and can account for the likely presence of inbreeding within clusters (given the selfing rate of *M. truncatula*). We used all polymorphic sites detected on control loci. Each site was considered as a single locus and we used a haploid setting. We ran Instruct, using Mode 1, which infers population structure only (no inference on the selfing rate) and assumes that admixture is possible between groups. For each run, we used a burn-in period of 5 × 10^5 ^iterations and then 10^6 ^iterations for estimating the parameters. Five independent chains were runs for each of 5 *K *values, *K *= 1 to *K *= 5. For each run, individual accessions were assigned into a group according to their highest proportion of membership into this group. The clusters thus defined were compared among runs for each *K *value and the best fitting K was chosen using the ΔK method of Falush *et al*. [32]. The fraction of the observed genetic variation explained by the inferred clusters was measured by the *F*_ST _parameter as implemented for nucleotide sequence data [33]. The stratification obtained for *K *= 2 appeared as the most robust one (see results) and was used in neutrality tests.

### Frequency spectrum neutrality test

We used patterns of polymorphism within each group to detect instances of selection causing departure from the neutral frequency spectrum. To do so we devised a test combining two neutrality test statistics relying on the underlying frequency spectrum: Tajima's *D *[8] and the standardized version of Fay and Wu's *H *[34,35] which we call Z. In order to obtain the joint distribution of these statistics under the hypothesis of selective neutrality while accounting for demographic history in each group, we followed the strategy used in De Mita *et al*. [23] and fitted population expansion models in each of the two groups. The procedure was repeated independently for each group and a different scaled mutation rate and rate of expansion were assumed for each group. We used an Approximate Bayesian framework [36] as implemented in the Python SeqLib library to obtain the joint posterior distribution for parameters.

We used the frequency of four minor allele frequency categories (0.125, 0.25, 0.375 and 0.5) as summary statistics of the data in each group. Then we approximated the joint posterior with 10^7 ^coalescent simulations and using local-linear regression on tan-transformed data with a tolerance factor of 10^-3^. The posterior joint distribution (scaled mutation rate and exponential population growth rate) was binned using 5184 evenly distributed bins over both dimensions. This posterior distribution was then used to obtain - through 10^6 ^independent simulations (by sampling a mutation and expansion rate to parametrize each coalescent simulation) - the distribution of *D *and *Z *expected at each locus. Note that locus specific sample size and alignment length were used in these simulations. A probability of misorientation of sites of 0.00936 was estimated from control loci [37] and incorporated in the simulations used to obtain the null distribution of (*D*, *Z*) at each locus. This was done by randomly switching the orientation (ancestral versus derived) of alleles after each coalescent simulation stage. This additional twist is an effective way to guard false positives, *i.e*. low p-values at locus evolving neutrally but where SNP misorientation is inflating Z. The (*D*, *Z*) distribution was discretized over 576 categories and the p-value of the bin containing the observed (*D*, *Z*) value of each locus in our data was computed.

### Evolutionary analysis of nucleotide substitution patterns

Patterns of between-species substitutions of *DMI1*, a gene previously identified as candidate for adaptation [23], were also analysed. To do this we sequenced most of the protein-coding sequence of *DMI1 *(same default protocol) in nine additional *Medicago *species (*M. ciliaris *[L0897E], *M. laciniata *[L0904E], *M. noeana *[L0908E], *M. polymorpha *[L0911E], *M. orbicularis *[L0913E], *M. sauvagei *[L0928D], *M. carstiensis *[LCarsX], *M. coerulea *[LCoerX], *M. ruthenica *[LRuth]) as well as previously cited *M. tornata*, *M. ciliaris*, *M. rigidula*, *M. rigiduloides*, *M. marina *and the L0736D accession from *M. truncatula*. In addition, we included the reference sequence *M. truncatula *Jemalong A17 from the literature (*DMI1 *mRNA sequence available as Genbank accession number AY497771).

Sequences were aligned at the protein level using MUSCLE version 3.8.31 with default options [38] with minor manual corrections to eliminate spurious amino acid replacements generated by the alignment procedure. For the same reason, the region spanning position 316 to 382 in the coding DNA sequence alignment (positions 280-303 in AY497771) consisting of gaps and poorly aligned sequences were removed. A molecular phylogeny was built with PhyML version 3.0 [39] using coding and protein sequences. We used the most conservative option of using the protein-based tree for further analysis as it minimizes the number of assumed amino acid changes. Using this phylogeny, we applied tests of positive selection based on codon-based substitution models [40] implemented in PAML version 4.2 [41]. The tests M2a versus M1a and M8 versus M8a yielded identical results and we report the first comparison only. We applied the test as recommended in PAML documentation and identified positively selected sites using a Bayes Empirical Bayes procedure.

## Results

### Levels of nucleotide variation in *Medicago truncatula*

**Figure 2:**
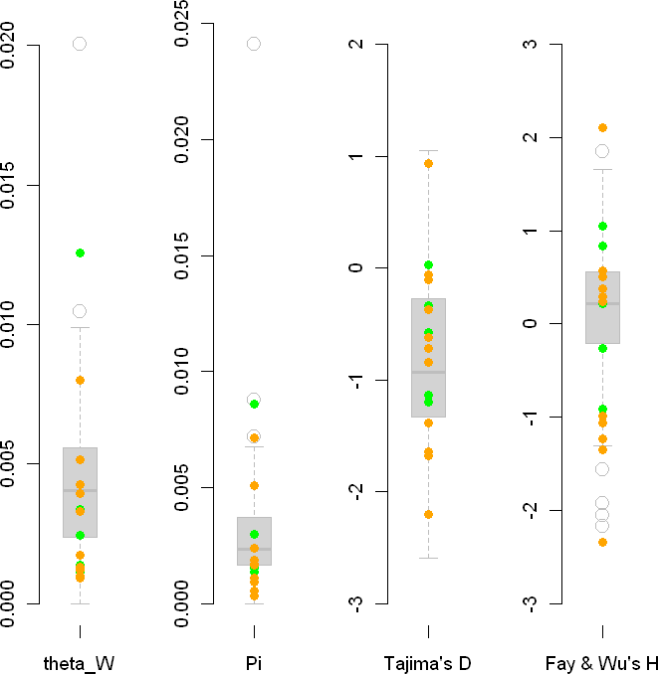
**Box plots summarizing patterns of nucleotide variation in *Medicago truncatula***. Boxplots (shaded gray) depict the empirical distribution obtained for control fragments. Dots represent individual flowering candidate genes (orange) and symbiotic genes (green). A: Distribution of the scaled mutation rate (as estimated with Watterson's θ) per bp for each fragment. B: Pairwise nucleotide diversity (π). C: Tajima's *D *statistic for each fragment. *Z*: standardized Fay and Wu's statistic.

We surveyed nucleotide diversity within *M. truncatula *by re-sequencing 53 gene fragments (37 control loci, 11 flowering-related and 5 nodulation-related). A total of 100,178 nucleotides were re-sequenced of which 74,233 were included in statistical analyses (after excluding sites with excessive missing data or alignment gaps). Gene fragments were sequenced in 57 *M. truncatula *accessions representing the whole-species diversity and in one outgroup. Overall *M. truncatula *is characterized by a fairly low level of nucleotide diversity at the species-wide level as measured through the scaled mutation rate (mean Watterson's θ = 0.0044 per base pair (bp^-1^) and mean nucleotide diversity is π = 0.0034, see Table 1), but in the range of studies surveying species with similar mating system: species-wide samples in *Arabidopsis thaliana *(θw ≃ 0.008 and π ≃ 0.006 [42]), in wild rice (0.0037 in *Oryiza rufipogon *[43]), and the self fertilizing worm *C. elegans *(π = 0.003 for silent polymorphism [44]) or within autogamous populations of *Eichornia paniculata *(π = 0.0008 [45]). Levels of nucleotide variation for control and candidate genes fragments are virtually identical (Figure 2). One notable exception is the nodulation gene *DMI3 *that exhibits very high levels of polymorphism (Table 1 and Additional file 4, Table S4).

**Table 1:** Summary of nucleotide polymorphism in control gene fragments

Average Statistic	Whole sample	Group 1	Group 2
Number of sequences used	50	22	28

Number of sites analysed	1208.27	1184.97	1217.70

Number of polymorphic sites	32.46	19.27	25.03

Watterson's θ^&^	0.004421	0.003231	0.003662

Nucleotide diversity (π)^&^	0.003409	0.002766	0.003185

Tajima's *D*	-0.81	-0.73	-0.58

Fu and Wu's *Z^#^*	0.04	0.03	-0.05

*F*_ST _between group 1 and 2	0.156	N/A	N/A

Hudson's ρ	0.063	0.020	0.164

All values are given as averages over the 37 control loci.

Group 1 and Group 2 are the two groups inferred using Instruct on sequence data obtained on control loci: Group 2 is the Spain-Morocco group and Group1 the eastern part of the sample (Figure 1)

&: per site estimates are reported here,

#: Z standardization of Fay and Wu's H.

N/A: not applicable.

The value Hudson's ρ reported here is the average of ρ estimates per analysed site.

Beyond average levels of polymorphism, we document a rather large variance in the scaled mutation rates as θ estimates span two orders of magnitude (from less than 0.0005 bp^-1 ^at *LEG722*, where no segregating sites were observed over 500 bp, to 0.02051 bp^-1 ^at *LAX4 *and 272 observed segregating sites over 8900 kb).

Overall, values for Tajima's *D *statistic in the whole sample vary widely but are generally skewed towards negative values (minimum -2.60, maximum 1.05, average -0.79, median -0.89). Candidate gene fragments and control fragment distributions are largely overlapping (Figure 2).

### The strength of purifying selection and adaptive evolution in *Medicago truncatula*

Next, we estimated the fraction 1-*f *of amino acid changing mutation undergoing strong purifying selection and the proportion α of non-synonymous divergence between *M truncatula *and our outgroup (*M. tornata*) that can be attributed to positive selection. We used 5 competing models - with varying amounts of purifying and positive selection - and their relative fit is summarized in Table 2. Model M0, which posits strict neutrality, fits the data poorly and was not considered further. Model comparison using differences in AIC provides suggestive evidence that, despite low level of polymorphism compatible with a rather small long-term effective size, a substantial amount of purifying selection constrains amino acid changing polymorphism. On average, about 90% of amino acid-changing mutations are removed by strong purifying selection. This is as strong or stronger than what has been reported for *A. thaliana *where 66% of amino-acid changing mutation where strongly deleterious [46].

**Table 2:** Intensity of purifying selection (1-*f*) and levels of adaptive evolution (α)

Model name and description	*f*	α	**Δ**_**AIC**_	**w**_**AIC**_
M0: Strict selective neutrality	1	0	321	~ 0

M1: Homogeneous purifying selection + No adaptive evolution	0.13	0	2.16	0.06

M2: Purifying selection with variable intensity + No adaptive evolution	*f*_1 _= 0.11 *f*_2 _= 0.17 *f*_3 _= 0.08	0	1.16	0.16

M3 Homogeneous purifying selection + homogeneous levels of adaptive evolution	0.11	0.36	0.79	0.24

**M4 Purifying selection with variable intensity + homogeneous levels of adaptive evolution**	***f*_1 _= 0.10** ***f*_2 _= 0.14** ***f*_3 _= 0.07**	**0.35**	**0**	**0.52**

M5: Purifying selection with variable intensity + varying levels of adaptive evolution	*f*_1 _= 0.09 *f*_2 _= 0.14 *f*_3 _= 0.09	α_1_ = 0.4 α_2_ = 0.37 α_3_ = -0.04	3.48	0.02

**Model average (over M0 to M5)**	***f*_1 _= 0.08** ***f*_2 _= 0.11** ***f***_**3 **_**= 0.06**	**α_1_ = 0.28** **α_2_ = 0.028** **α_3_ = 0.027**	**N/A**	**N/A**

1-*f *quantifies the intensity of purifying selection through the fraction of new amino acid changing mutation under strong purifying selection. α is the fraction of divergence attributable to adaptive evolution (driven by positive selection on amino acid changing mutations).

The best model (M4) is highlighted in bold and all differences in AIC among models Δ_AIC_) are computed relative to M4. W_AIC _are the statistical weights of each model in the model averaging procedure we used.

Subscripts in *f*_1_, *f*_2_, *f*_3_, α_1_, α_2_, α_3 _refer to the gene fragment categories. 1: control fragments, 2: flowering gene fragments, 3: symbiotic gene fragments. N/A: not applicable.

The estimated level of adaptive evolution is high (α ≃ 0.25) but there is a very large sampling variance and this estimate is not statistically significant from zero (likelihood ratio test comparing M2 versus M4 = 3.2, *p = *0.07). Last, we asked if a model (M5) positing that different types of gene fragments experienced different level of adaptive substitution improved the fit of the data. Comparison with a model with homogeneous level of adaptive substitutions (M4) suggests that flowering genes may experience more adaptive substitution (α = 0.37) and that symbiotic genes experience fewer substitution (α = -0.04) but again sampling variance around the estimates of α for each category of genes is very large and the model M5 overall fits poorly the data relative to a more parsimonious one (M4). We thus choose to report more robust model averaged estimates of *f *and α in Table 2.

### Broad patterns of genetic subdivision in *Medicago truncatula*

We studied broad patterns of genetic subdivision in our sample and inferred stratification in *K *groups within our species-wide sample. We used values of *K *ranging between 1 and 5. Consistent estimates of log-likelihood and stratification patterns were obtained for all *K *values. Posterior probabilities of the data always increased with increasing subdivision (*i.e*. higher *K*); the highest gain occurring between *K *= 1 and *K *= 2 and between *K *= 2 and *K *= 3 (Additional file 5, Figure S1).

At *K *= 2, the inferred structure was stable across runs, geographically consistent, and in agreement with a previous structure inferred on the basis of SSR variation in a set of 13 microsatellite loci on a much larger sample (*n *= 347) of accessions [20]. This stratification splits our species-wide sample into a group of 32 accessions originating from the South-West of the Mediterranean basin and a group of 25 accessions originating from a broad Eastern part of the Mediterranean basin (Figure 1). Except for three accessions (L00213, L00368 and L00401), all the accessions in this study were assigned to the same group as when using SSR data. This stratification accounted for 17% of the total genetic variability in our data. At *K *= 3, independent runs were also consistent, but the stratification obtained was not geographically consistent, splitting the two groups defined at *K *= 2 with no clear pattern (Additional file 6, Figure S2). This stratification also explained less genetic variability than *K *= 2. For *K *= 4, the stratification was the same as that obtained with *K *= 3 except for one individual (L00213) which constituted alone the fourth group. At *K *= 5, most accessions appeared admixed, *i.e*. showing proportions of membership into each of the 5 clusters lower than 0.3 or 0.4.

Given the geographic consistency of the subdivision obtained at *K *= 2 and the congruence of this subdivision with that obtained using SSR loci, we choose to use *K *= 2 as the subdivision pattern for further analyses. Note that this analysis is by no means intended to infer the actual number of populations underlying our sample but merely a way to group accessions in fairly homogeneous groups. The two groups show relatively similar levels and patterns of nucleotide variation (Table 1) despite the lower geographic area covered by the Western group (referred as group 2). Both groups also exhibit negative Tajima's D values, suggesting recent events of population expansion occurred in both groups (see Additional file 7, Table S5 for details on diversity statistics per fragment).

### The frequency spectrum at several flowering genes departs from neutrality and suggests recent or ongoing selection

To determine if patterns of polymorphism departed from selective neutrality we combined two statistics, *D *and *Z*, summarizing the frequency spectrum. This level of analysis strikes a compromise between retaining high enough polymorphism while using a sample for which we can model patterns of polymorphism (in order to obtain null distributions for neutrality test statistics). Our approach builds on previous work showing that a scenario assuming a single population with exponential growth, although simplistic, can adequately capture patterns of polymorphism in control gene fragments [23].

**Figure 3:**
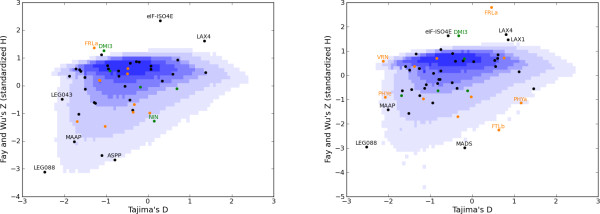
**Tests of selective neutrality of polymorphism within each group**. The joint distributions of (*D*, *Z*) tests statistics expected under neutrality in the Eastern group (group 1, panel 3a) and the Western group (group 2, panel 3b) are plotted using a blue shading for the probability density. Symbiotic genes are plotted as green dots and flowering genes as orange dots using each candidate gene abbreviation. Control loci are represented as black dots. The joint distribution for (*D*, *Z*) within each group was obtained through 10^5 ^coalescent simulations from models parametrized to fit patterns of polymorphisms in the set of controlled fragments (See Methods for further details). Note that in order to generate a unique graphical representation of the neutral joint distribution, simulations for each of the 53 loci were pooled and the resulting (*D*, *Z*) distribution was binned using 10^4 ^categories (rigorous p-values computed using null distributions tailored for each locus length and polymorphism are available in Additional file 7, Table S5).

We mapped the observed values (*D*, *Z*) of each gene fragment on the joint density of (*D*, *Z*) expected under selective neutrality while accounting for the demographic history of each group (see methods for details). A graphical summary of our results is presented in Figure 3 (p-values are in Additional file 8, Table S6). P-values of the joint test (*D*, *Z*) obtained in the two groups exhibit virtually no correlation (observed correlation of 0.12 across *n *= 46 loci, *p *> 0.4). Empirical distribution of p-values at control loci was also slightly L-shaped (not shown), suggesting that despite our efforts to account for the coalescent history of our sample, our test is still probably slightly too liberal so that p-values should be interpreted with caution. Among the 16 surveyed candidate genes, observed patterns of nucleotide diversity (as summarized by values of *D *and *Z*) fall outside the simulated neutral distribution for 7 fragments: *FRLa *and *DMI3 *exhibit *Z *values in both groups that are larger than expected under neutrality, *FTLb *and *PHYa *exhibit too negative Z values in the Western group 2, *VRN *and *PHY*e exhibit too negative *D *values in the (Western) group 2, and *NIN *exhibits a negative Z value in the Eastern group (group 1). Several loci - including control loci - exhibited low p-values and inspection of the (*D, Z) *coordinates reveals loci with abnormally high values of *Z *but typical values of *D*. Negative values of *Z *sign an excess of high-frequency derived mutations, which can be interpreted as hitch-hiking episodes driven by recent selection. In contrast, occurrence of positive *Z *values (deficit of high-frequency derived mutations) cannot be explained by natural selection in any straightforward manner. Notably, genes displaying high values of *Z *often exhibit the highest diversity indices (sometimes by orders of magnitude compared to other genes). *Z*, unlike *D*, might be sensitive to variations in mutation rate. Even worse, *Z *is very sensitive to variation of gene genealogy structure, as deep genealogies might pre-date the divergence with outgroups and drive spuriously high values of *Z*. As a result, we chose not to report as departing from neutrality loci exhibiting a very high value of *Z *and a high diversity, as for these an artefact due to high mutation rate seems likely (see below how sensitivity of *Z *in the [*D*, *Z*] test could be addressed).

Barring such cases, this method detected footprints of selection in four genes potentially involved in flowering date (*FTLb*, *PHYa, VRN and PHYe*) and one symbiotic gene (*NIN*). The gene *FTLb *displayed in the Western group a very negative *Z *value compared to neutral expectations, suggesting positive selection on this gene while *PHYa *exhibited a negative *Z *value but also a strikingly large *D *value in this group, as expected under diversifying selection or local adaptation. Both *VRN *and *PHY*e, displayed very negative *D *values as expected under directional selection. For these two genes, however, the deviations from expected values were less important resulting in larger p-values (*p *= 0.025 and 0.023 respectively compared to 0.0066 for *FTLb *and 0.0006 for *PHYa*). *NIN *exhibited a negative *Z *value as expected under positive selection in the Eastern group only (*p *= 0.019).

### Evidence for selection in *DMI1*

**Figure 4:**
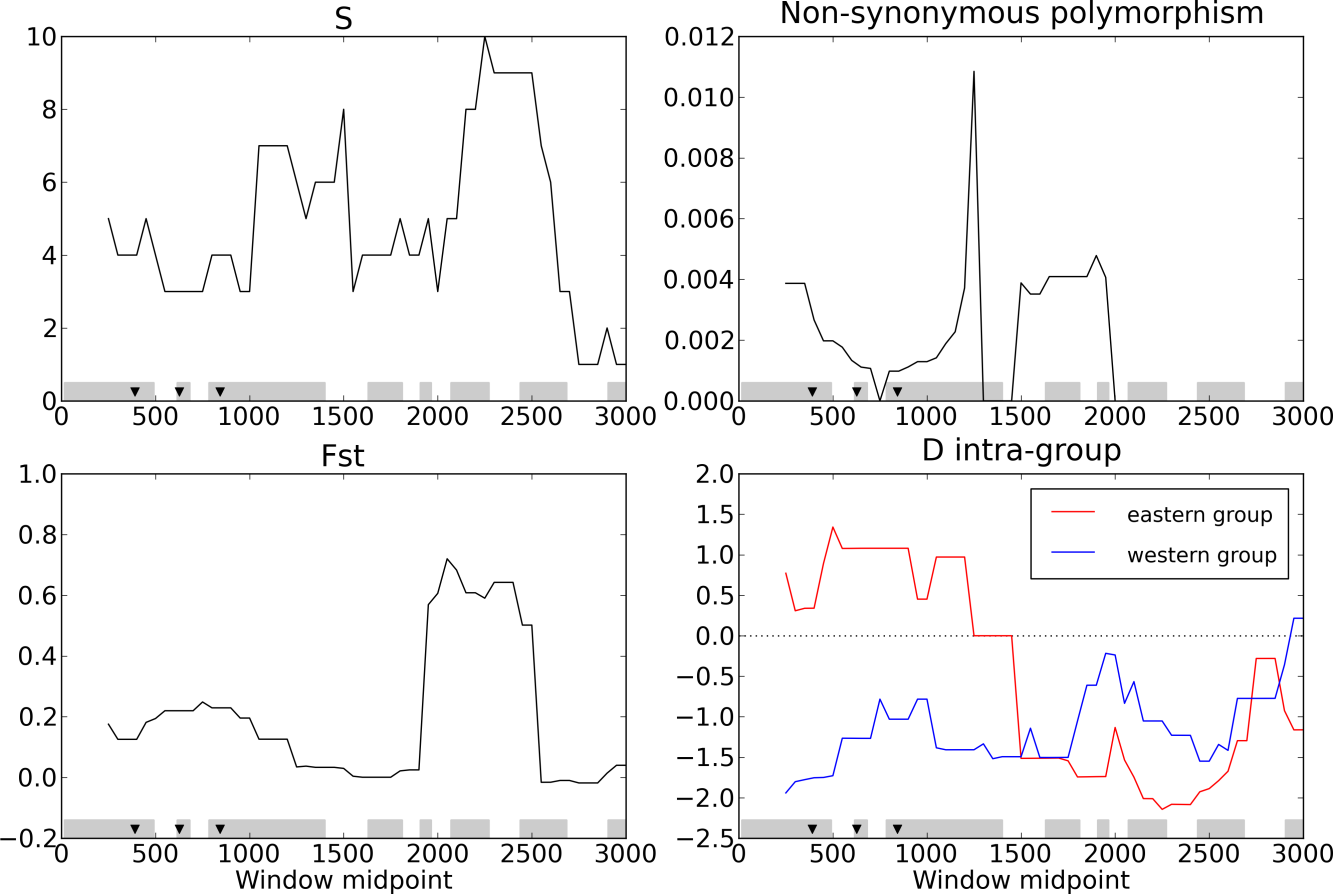
**Sliding window analysis of the gene *DMI1***. Polymorphism was analysed in 500 nucleotide-long windows with 50 nucleotide steps along the alignments. Displayed statistics are: *S*, the number of polymorphic sites per window, non-synonymous polymorphism given by Watterson's θ per non-synonymous site, *F*_ST _given by Weir and Cockerham's estimator [59] and Tajima's *D *computed in both groups. The grey frames denote the positions of exons and the arrowheads mark the position of the sites found with high posterior probability (> 0.95) to be targets of positive selection in the *Medicago *genus.

*DMI1 *was the only nodulation candidate detected in a previous analysis [23] but is not singled out in the current application of the (*D*-*Z*) test. However several SNPs located in exon 6 of *DMI1 *exhibited an excess of differentiation. The analysis of local variation of patterns of polymorphism shows interesting features (Figure 4), although challenging to interpret. The strikingly contrasted pattern of differentiation and Tajima's *D *might explain why this gene could not be detected using gene-wise summaries of polymorphism and suggest that selection occurred in the gene (see the Discussion section for possible interpretations). Codon evolution analysis strongly supports the hypothesis that several sites in *DMI1 *evolved under positive selection in the history of the *Medicago *genus. A model allowing for positive selection on a fraction of the sites (M2a) fitted much better the data than a model (M1a) with no sites under positive selection (likelihood ratio test, *p *= 0.016). The location of the sites that are very likely to have evolved under positive selection is represented in Figure 4.

## Discussion

### *M. truncatula *nucleotide polymorphism is shaped primarily by purifying selection

The species-wide levels of nucleotide polymorphism we report here are in the lower range of plant studies which is consistent with the expectation that selfing plant species harbour less nucleotide diversity than outcrossing species [47]. Note however that although mating system was the variable explaining most of the variation in polymorphism across plant species, the portion of the total variance in nucleotide diversity accounted by mating system alone is rather modest (less than 10% after phylogenetic relatedness is accounted for). Levels of polymorphism in *M. truncatula *thus lie in the low range although these are by no mean as extreme as the striking instance of some self-fertilizing species - *Capsella rubella *that recently evolved from the outcrossing *Capsella grandiflora *- which only displays one to two haplotypes throughout its species range [48,49]. We also report fairly strong purifying selection removing up to 85% of all non-synonymous (amino acid changing) mutations and marginal evidence for heterogeneity in the amount of purifying selection and positive selection among gene categories. Although the evidence for positive selection on flowering genes obtained by contrasting polymorphism and divergence (Table 2) is suggestive and also consistent with evidence obtained on a different time scale using the (*D*, *Z*) test, estimates of α should be viewed as preliminary for two reasons. First, α estimates are inherently noisy [9] and data on many more genes for each category would be needed to reduce sampling variance around estimates. Second demography is expected to affect the estimation of both *f *and α [29].

### Model-based tests of selection

Instead of relying on non-parametric methods for detecting outliers suggestive of selection, we employed model-based methods. These rely on first fitting patterns of nucleotide variation at a set of control loci as a means of parametrizing a model positing selective neutrality and the most salient features of the demographic history that may be responsible for the patterns of nucleotide diversity in our sample. We discussed below insights provided by these approaches but also their limits.

Some loci, including controls, displayed large values for Fay and Wu's standardized *H *statistic (Z) that were strongly unlikely under our model. These genes also ranked among those with the highest per-site Watterson'sθ. While *D *is virtually insensitive to levels of polymorphism, *Z *and even more the non-standardized *H *statistic are dramatically so. Allowing for variation of the mutation rate among genes would make the (*D*, *Z*) test more robust. One option is to constrain θ to be proportional to an independent estimate of mutation rates or a proxy such as interspecific synonymous nucleotide divergence. The outgroups we used were however too close - in terms of absolute amount of divergence to our sample - for this purpose, yielding putatively very noisy estimates for mutation rates that would exacerbate rather than fix the problem. At the cost of extra parameters, the variation of θ among genes could be treated as a hidden statistical parameter (such as the variance of a statistical distribution) to be estimated.

Our model for patterns of polymorphism within group, by ignoring gene flow between geographic regions comprising each group of accessions, underestimates the variance in coalescence times underlying amounts of polymorphism. Thus loci with neutral polymorphism but high θ and in turn possibly high *Z *values were picked as departing from neutrality. A more sophisticated model would treat both populations jointly but addressing this would require a flexible yet computationally tractable population structure component for coalescence models. That remains a theoretical challenge.

We observed a broad population structure (*K *= 2) consistent with previous analysis of SSR data [20] which, in the absence of obvious barrier to gene flow, seems likely a relic of past isolation reflecting the existence of glacial refuges both West (Spain) and East (Levant) of the Mediterranean basin. Interestingly clear footprints of selection were detected only in one of the two groups (the western group 2) suggesting that selective pressures differ across Western and Eastern parts of the species range. No obvious environmental differences between these two geographic regions could be identified: both regions span a similar latitudinal gradient and share the mosaic of climate, geology and anthropic pressures that characterize the Mediterranean [50]. The only notable difference between these two groups is the size of the geographic area they span, the Western group being restricted to the Iberian Peninsula and Northern Morocco.

### Molecular adaptation in the nodulation and flowering pathway

Although the majority of *Arabidopsis *flowering genes have orthologs in legumes, little is known about the genes underlying variation in flowering time in *Medicago truncatula*. Previous studies have shown that as most Mediterranean legume species, *Medicago truncatula *exhibits reduced time to flowering under vernalization and under long day conditions [51]. We studied orthologs of genes that have been shown to be involved at different levels of the flowering signalling pathway in *Arabidopsis thaliana*: *PHYa*, *PHYb*, *PHYe*, *VRN *and *FCA *are involved in the perception of environmental stimuli, *COL (Constans-Like) *and *FRLa *(*Frigida-Like*) are central genes and *FTLa*, *FTLb*, *FTLc *and *SOC1b *are integrating genes [52]. We detected several signatures of selection in genes involved in the perception of environmental stimuli (*PHYa*, *PHYe *and *VRN*). Phytochromes are a family of plant photoreceptors that mediate physiological and developmental responses to changes in light conditions. In *Arabidopsis thaliana*, five phytochrome (*PHYa-e*) genes have been shown to be involved in the timing of flowerng, with *PHYa*, *PHYd*, and *PHYe *playing a major role in the control of flowering under cool conditions [53]. *VRN1 *and *VRN2 *have been shown to mediate response to vernalization both in *Arabidopsis thaliana *and cereal crops [54].

We note that most footprints of selection detected in this study involve the most upstream genes of the flowering pathway. Such genes are expected to be associated with lower pleiotropic effect and are thus expected to be preferential targets of natural selection [55]. We also detected a significant deviation from neutral expectations on the integrator gene *FT *(*FLOWERING TIME*). This gene acts on the floral meristem identity genes to initiate flowering and is one of six candidate genes that have been shown to be located in the support interval of a QTL that explained 10 to 60% of the flowering date variation in three RILs populations [56].

Further phenotypic and climatic data are needed to evaluate the role of these different genes in natural variation for flowering date in *Medicago truncatula *and to understand their involvement in the adaptation of the species to climatic variations.

For nodulation genes, we detected footprints of selection in two genes *DMI*1 and *NIN*. Both genes are acting at the signal transduction level, without direct interaction with the symbiotic partner. *DMI*1 appears to have undergone selection both recently - between *M. truncatula *populations - and more anciently in the *Medicago *genus. A sliding window analysis reinforces this statement (Figure 4) while similar analyses for other candidate genes did not uncover obvious local signatures of selection along the gene (data not shown).

## Conclusion

We used Sanger sequencing to survey nucleotide variation in *Medicago truncatula*. We focused on a set of important genes with known function in the Nod factor perception and flowering pathways and asked if selection had affected these genes at different evolutionary time-scales. We did so by contrasting patterns of polymorphism at candidate genes with a set of control gene fragments and a coalescent framework where simple demographic models were fitted to the control data. The fitted demographic models were then used as a basis to obtain - via coalescent simulations - null distributions for statistics characterizing patterns of variation in genes of interest. Here we uncover several footprints of selection in both pathways and at different evolutionary time scales. Our detailed analysis of diversity within groups demonstrates that model-based methods of analysis can be quite sensitive to assumptions of the underlying models used. Future models should explore more detailed demographic scenarios, possibly in a more spatially explicit context (see for instance [57] for analysis of spatial patterns of polymorphism in *A. thaliana*). Inspection of the empirical distribution of scaled mutation rates also suggests ample variation in per nucleotide mutation rate throughout the genome. Even if neutrality test statistics are built in order to minimize sensitivity to underlying variation in mutation rates, incorporating genomic heterogeneities in mutation and recombination along the genome may be needed to generate sound null distributions that integrate over both historical and mutational/recombinational unobserved processes that shape genomic diversity [58]. Fitting such models requires orders of magnitude of more polymorphism data but these will be available in the near future for the *M truncatula *species complex [http://medicagohapmap.org/] and increasingly so for non model species. Thus, barring the computational challenges to "scale up" to genome levels, model based approaches incorporating the additional touch of biological realism described above will be a fruitful framework for understanding evolutionary forces shaping genome variation and, ultimately, symbiotic evolution and flowering time in this system.

## Availability of supporting data

The data sets supporting the results of this article are available in the Dryad repository at doi:10.5061/dryad.9031.

## Authors' contributions

TB and JR designed and coordinated research. SDM, NC, KL performed sequencing, alignment and annotation. SDM, JR, TB analyzed data. SDM TB and JR wrote the paper. All authors read and approved the final manuscript.

## Supplementary Material

Additional file 1 **Table S1 - List of *Medicago truncatula *accessions**. Microsoft Word document containing the list of *Medicago truncatula *accessions that were re-sequenced in this study, their accession number, country of origin and sample information. Click here for file

Additional file 2 **Table S2 - PCR primers**. Microsoft Excel spreadsheet containing the list of PCR primers used to amplify and sequence flowering and symbiotic gene fragments. Click here for file

Additional file 3 **Table S3 - List of sequenced loci**. Microsoft Excel spreadsheet containing the list of sequenced loci, genomic location and results of sequencing. *la *is the alignment length and *ls *is the length of the sequenced obtained from A17 with alignment gap stripped. Click here for file

Additional file 4 **Table S4 - Standard diversity indices for each fragment**. Microsoft Excel spreadsheet containing standard diversity indices computed for each fragment. The results of different summary statistics are presented in separate sub-tables for control fragments, flowering loci and nodulation loci. NC: not computed. Click here for file

Additional file 5 **Figure S1 - Likelihood profile for *K*, the number of groups in the sample**. Microsoft Word document showing the likelihood profile for *K *in the structure analysis. Likelihood was obtained using the software Instruct on the nucleotide polymorphism data from control loci. Click here for file

Additional file 6 **Figure S2 - Group membership of the accession in structure analysis**. Microsoft Powerpoint presentation containing the schematic representation of the assignment of the accessions in each group following Instruct analysis for *K *= 2, *K *= 3 and *K *= 4. Click here for file

Additional file 7 **Table S5 - Standard diversity indices for each fragments within both ancestry groups**. Microsoft Excel spreadsheet containing the values of **s**tandard diversity indices estimated for each fragment within both ancestry groups. NC: not computed. Click here for file

Additional file 8 **Table S6 - P-values of the joint (*D*, *Z*) test**. Microsoft Excel spreadsheet containing, for each locus, the value of *D*, *Z*, and the p-value for each group (P1 and P2 are the two p-values for a given locus in group 1 and group 2 respectively). Click here for file
