# Supplementary material for: Molecular adaptation in flowering and symbiotic recognition pathways: insights from patterns of polymorphism in the legume Medicago truncatula
Source: BMC Evol Biol. 2011 Aug 1;11:229. doi: 10.1186/1471-2148-11-229 (PMC3199773; doi:10.1186/1471-2148-11-229)
Supplement: Additional file 1 — Table S1 - List of Medicago truncatula accessions. Microsoft Word document containing the list of Medicago truncatula accessions that were re-sequenced in this study, their accession number, country of origin and sample information. [file 1471-2148-11-229-S1.DOC]

## Supplementary Table 1: List of genotypes resequenced

| **Name** | **Accession label** | **Country of origin** | **Sample^(1)^** |
| --- | --- | --- | --- |
| SA.09707 | L00049 | Tunisia | CC32 |
| SA.14163 | L00144 | Jordan | CC32 |
| SA.24714 | L00154 | Italie | CC32 |
| SA.22322 | L00163 | Syria | CC32 |
| SA.25654 | L00166 | Morocco | Regional Group |
| SA.28064 | L00174 | Cyprus | CC32 |
| SA.09048 | L00198 | Libya | CC32 |
| SA.27882 | L00213 | Morocco | CC32 |
| SA.27961 | L00216 | Morocco | Regional Group |
| SA.24576 | L00233 | Morocco | Regional Group |
| SA.26063 | L00239 | Morocco | CC32 / Regional Group |
| SA.14161 | L00245 | Jordan | CC32 |
| SA.03116 | L00263 | Israel | CC32 |
| SA.09119 | L00290 | Turkey | CC32 |
| SA.08623 | L00306 | Morocco | Regional Group |
| SA.09944 | L00310 | Tunisia | CC32 |
| SA.03780 | L00321 | Italie | CC32 |
| SA.02806 | L00330 | Portugal | Regional Group |
| GRC042-1 | L00337 | Greece | CC32 |
| DZA202-4 | L00357 | Algeria | Regional Group |
| DZA012-J | L00368 | Algeria | CC32 |
| PRT180-A | L00369 | Portugal | CC32 / Regional Group |
| PRT177-C | L00372 | Portugal | Regional Group |
| DZA323-3 | L00400 | Algeria | Regional Group |
| ESP031-A | L00401 | Espagna | Regional Group |
| ESP039-A | L00404 | Espagna | Regional Group |
| ESP043-B | L00410 | Espagna | Regional Group |
| ESP050-B | L00414 | Espagna | Regional Group |
| ESP098-C | L00421 | Espagna | Regional Group |
| ESP100-G | L00425 | Espagna | Regional Group |
| ESP104-A | L00430 | Espagna | Regional Group |
| ESP175-D | L00448 | Espagna | Regional Group |
| ESP095-C | L00482 | Espagna | Regional Group |
| ESP163-C | L00514 | Espagna | Regional Group |
| PRT179-F | L00526 | Portugal | Regional Group |
| F83005-5 | L00530 | France | CC32 |
| DZA233-4 | L00542 | Algeria | CC32 |
| DZA327-7 | L00543 | Algeria | CC32 / Regional Group |
| ESP105-L | L00544 | Espagna | CC32 / Regional Group |
| ESP158-A | L00545 | Espagna | CC32 / Regional Group |
| ESP159-11 | L00546 | Espagna | Regional Group |
| ESP165-D | L00547 | Espagna | Regional Group |
| F11005-E | L00549 | France | CC32 |
| F11013-3 | L00550 | France | CC32 / Regional Group |
| F20047-A | L00552 | France | CC32 |
| F20089-B | L00554 | France | CC32 |
| GRC020-B | L00555 | Greece | CC32 |
| GRC064-B | L00557 | Greece | CC32 |
| DZA213-K | L00640 | Algeria | Regional Group |
| salse42B | L00648 | France | CC32 / Regional Group |
| salse71B | L00651 | France | CC32 |
| DZA319-- | L00673 | Algeria | Regional Group |
| F66017-- | L00679 | France | CC32 / Regional Group |
| DZA315-16 | L00734 | Algeria | CC32 |
| DZA045-6 | L00736 | Algeria | CC32 |
| A20 | L00732 | unknown | reference |
| A17 | L00738 | unknown | reference |

(1) this column gives the repartition of the accessions in the two hierarchical samples studied : the species level was represented by the *CC32* sample, the regional scale by the ‘Spain-Morocco’ group (*Regional group*)*.*
