# Supplementary material for: Molecular adaptation in flowering and symbiotic recognition pathways: insights from patterns of polymorphism in the legume Medicago truncatula
Source: BMC Evol Biol. 2011 Aug 1;11:229. doi: 10.1186/1471-2148-11-229 (PMC3199773; doi:10.1186/1471-2148-11-229)
Supplement: Additional file 6 — Figure S2 - Group membership of the accession in structure analysis. Microsoft Powerpoint presentation containing the schematic representation of the assignment of the accessions in each group following Instruct analysis for K = 2, K = 3 and K = 4. [file 1471-2148-11-229-S6.PPT]

## Slide 1
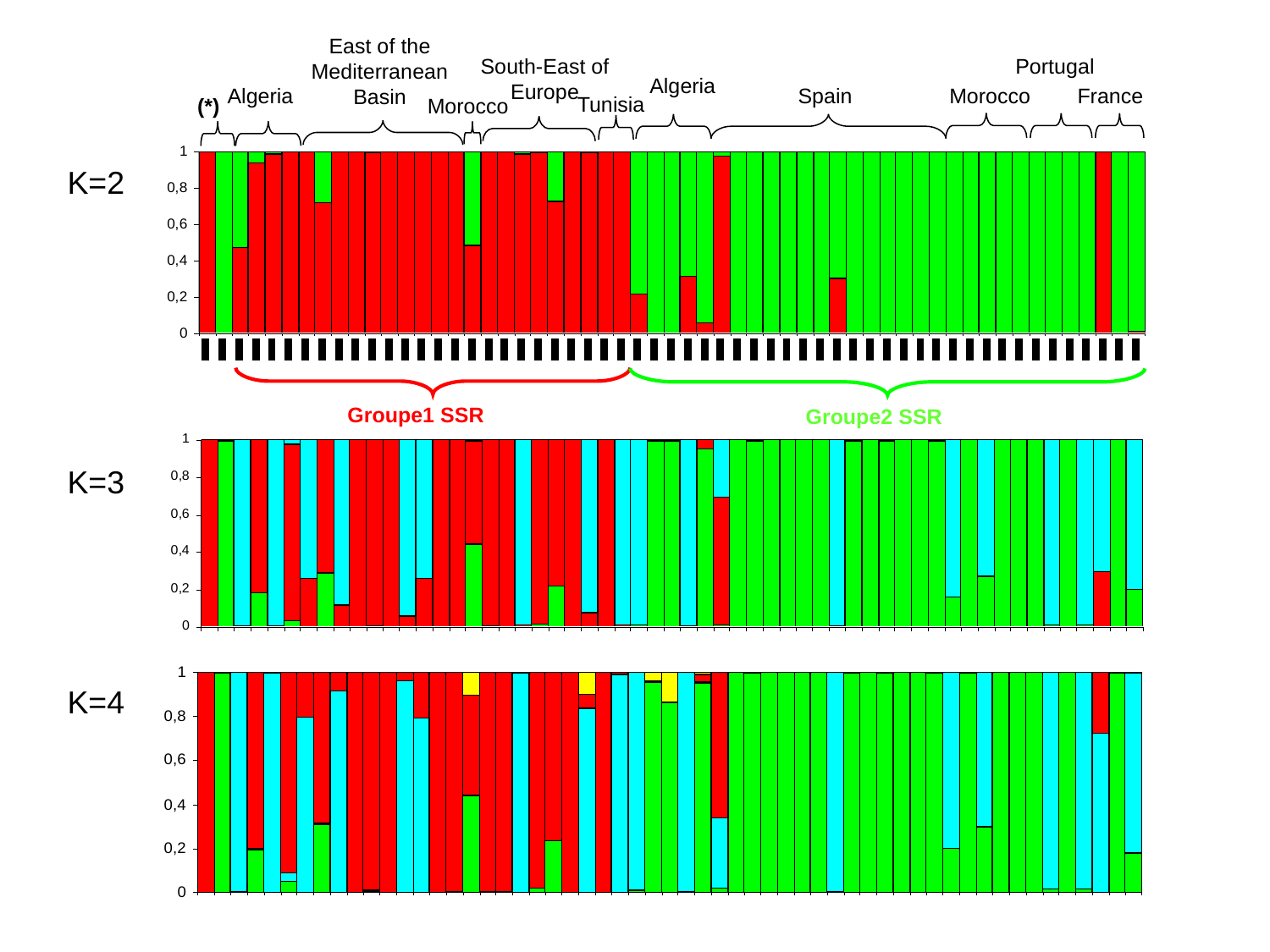

East of the Mediterranean Basin
South-East of Europe
Portugal
Algeria
Algeria
Spain
Morocco
France
Tunisia
(*)
Morocco
K=2
Groupe1 SSR
Groupe2 SSR
K=3
K=4
