# Supplementary material for: Molecular adaptation in flowering and symbiotic recognition pathways: insights from patterns of polymorphism in the legume Medicago truncatula
Source: BMC Evol Biol. 2011 Aug 1;11:229. doi: 10.1186/1471-2148-11-229 (PMC3199773; doi:10.1186/1471-2148-11-229)
Supplement: Supplementary file 6 [file 1471-2148-11-229-S5.DOC]

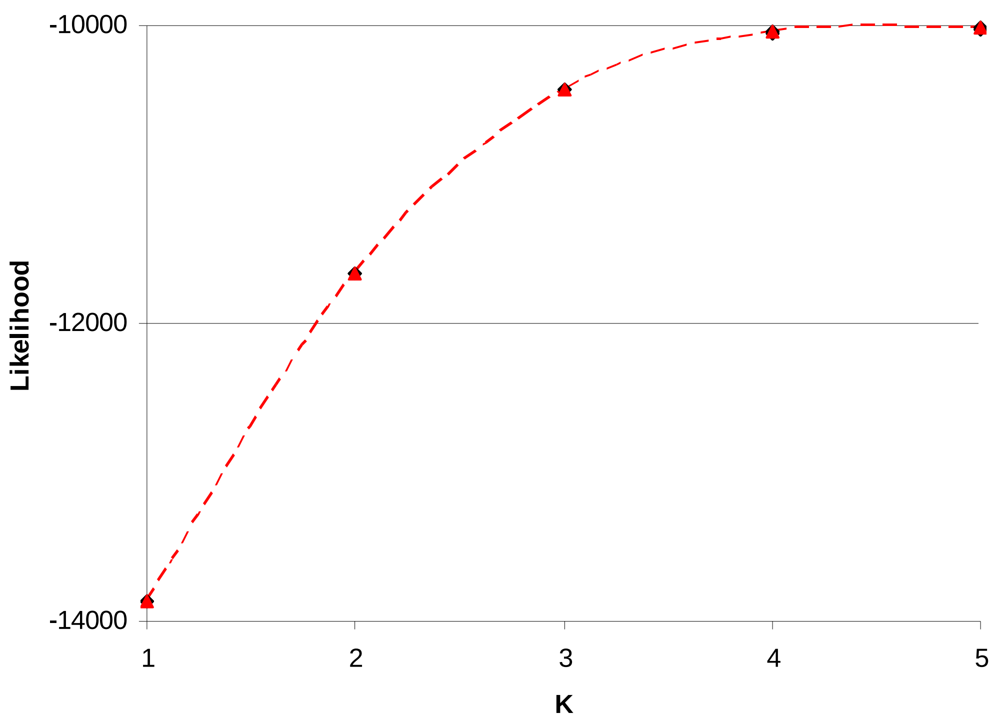

-10000
-12000
-14000
Likelihood
1
2
3
4
5
K
